# Epulis

**Published:** 1863-10

**Authors:** Leon. F. Harvey

**Affiliations:** Buffalo, N. Y.


### DENTAL REGISTER OF THE WEST

VOL. XVII.] OCTOBER, 1863. [NO. 10.

### Original Essays and Communications.

### EPULIS.

BY LEON. F. HARVEY, M. D., BUFFALO, N. Y.

Read before the Western N. Y. Dental Association.

MR. PRESIDENT AND GENTLEMEN.—We purpose calling your attention for a few minutes to some remarks upon the subject of Epulis. Epulis from επι "upon" and ουλον "the gums." This variety of tumor we find frequently in the practice of dentistry, and it should not be left to the surgeon to treat, but let the dentist attend to it at once, and that in such an efficient manner, that a second operation will not be required, for they are often removed for the third and fourth time. We shall recognise two classes, the malignant and non-malignant. The latter is most frequent and of that we will now speak. This tumor is generally fibrous in character, most often appearing upon the lower jaw, though sometimes it is found upon the upper. It is nearly always attached to the alveoli, and grows between the teeth nearly or quite to their grinding surfaces. If allowed to progress very far, the teeth involved are loosened. In appearance it is of a reddish or reddish blue color, smooth though lobulated. It quite firm till ulceration takes place, and it softens and a discharge of pus ensues. This form of tumor occurs in persons of all ages, though more frequently with the young. They may arise from caries of the teeth or an abrasion of the gum. If removed effectually they are not reproduced.

Malignant Epulis occurs generally in persons at an advanced age. The tumor is of a purplish or violet color—is more often soft than hard. By some authors they are described as hard, rough and schirrous Epulis. A bloody discharge generally issues from them—are very indolent in their growth—the pains are sharp and lancinating, being more frequent at night. They take their growth in the same manner as the non-malignant, are filled with vessels, and after a certain time are rapid in their growth, and appear again after removal. The causes of this variety are the same as the other, with the addition of the cancerous cachexia. A non-malignant Epulis may resolve itself into a malignant, if allowed to become irritated, and treatment the most vigorous is not adopted early. Again, if not removed, the osseous portion of the jaw may become deeply involved, and a removal of it become necessary. The time at which the malignant form destroys life varies from one to three years. Saurel in a Memoir on Tumors of the Gums, known as Epulis, published in Vol. 8, New Series, of the American Journal of Dental Science, says: " the structure of the tumors of the gums has hitherto been very little studied. I have found anatomical characters given in only four reported cases, so that in spite of the microscopic labors of M. Robin, this part of their history is still a desideratum. Finally, considering all the data possessed by science and reviewing the clinical study, I have undertaken in the second part of this work, I consider myself authorized to admit the following species:—

1. Simple epulis, consisting of a hypertrophy or abnormal development of the proper tissue of the gums.

2. Fungus epulis, characterized by a production of fungositus or fleshy vegetations, flabby, spongy, shaped like a mushroom composed of a great number of vessels and of fibro-plastic tissue.

3. Vascular or erectile epulis "the organization of which appears to be the same as that of erectile tumors."

4. Fibrous, fibro-plastic or fibro-cartilaginous epulis composed of a white, compact, incompressible tissue, not capable of being crushed, affording no cancerous juice, but little vascular, with resisting fibres or lamella intersecting one another in all directions. It is composed chiefly of fibrous and fibro-plastic elements.

5. Bony epulis. In the single case of this variety with which we are acquainted, the tumor was composed entirely of bony tissue with large meshes. Other fibrous or carcinomatous tumors contain nullei or concretions of bone—which seems to establish a sort of relation among the different species of epulis.

6. Carcinomatous epulis. This species originates in the bony tissue which it destroys, and for which it substitutes itself, we have found it formed of a dense cartilaginiform tissue resembling lardaceous cancer, and bony in several points, especially towards the central parts. These bony concretious are met with only in the free portion of the tumour; that which is implanted in the bone, is composed of a soft tissue which stimulates its absorption, and leads to the formation of little spiculæ. This has imposed upon certain surgeons by leading them to suppose that the disease depended upon a caries or menus of maxillary bones. It is to these tumors that the microscopical characters described by M. Robin belongs."

There are three methods of treatment—two described in the books. The first is the removal of the teeth on either side of the tumor, and then with the saw cutting down upon the jaw to the base of the tumor and that portion removed. This is an operation hardly warranted as one or more sound teeth are necessarily lost, but should the teeth have become very loose, and it is clearly seen that they will never be firm, it may be well to remove them, but we should hardly consent to the removal of the bone—for the loss of the teeth will occasion enough falling in of the face, but the removal of a piece of bone would cause a deformity which would not be justifiable.

The second mode of treatment consists simply in the removal of the tumor with the knife, and scraping all the periosteum from the alveoli and the use of caustic. Frequently cutting or tearing the tumor off, will accomplish the object sought, but these as two cases in illustration will show, must have a very small pedicle. Regin thus speaks of an erectile epulis:—" a young officer who came to consult us from the Val-de-Grace, had for some months an epulis, whose size equalled the egg of a pigeon, situated upon the internal side of the right branch of the inferior maxiliary, which covered in some degree the tongue, and thus impeded its movements. By examining the tumor, I found it supported by a pedicle so straight and so yielding, that by passing my finger under it so as to form a crotchet, I broke it without an effort and it fell from the mouth, after a considerable flowing of blood the patient departed, and experienced no relapse."

Ferguson, at page 511 of his Surgery, says:—Once I saw a tumor of this kind of the size of a walnut connected with the gum within the incisor teeth of the lower jaw, the patient had gone near her full time with child, and I did not, therefore, advise its immediate removal, although this might have been read.ly accomplished as it was attached only by a small neck, in the course a few weeks after she rubbed it off with the point of her tongue, and was never further troubled." Cases of epulis seldom terminate so favorably, however, and some more energetic means, than the female tongue, must be resorted to.

The third operation, not found in the books, and which we do not remember as having been advised by any one—is as follows: the tumor is torn from its attachment, the bone removed by little chips, and caustic applied. Ambrose Pari, speaks of having seen one of these excrescences, so volum-

ınous, that part of it appeared without the mouth and rendered the patient a hideous object. He says he destroyed them by first tying them with a very strong double thread and afterwards by applying an actual cautery, in order to destroy the remaining part of the tumor. He adds that this tumor is often cartilaginous, and that even in time becomes bony. We shall here give an account of two operations, the first by Saurel; the tumor removed by him was a fibrous one, the patient being seated in front of a window, the tumor was dissected and removed with a bistoury. I took care to go beyond its base and act upon the sound portion of the gum; a little blood ran from the lower portion of the wound; this hemorrhage yielded to a light compression by the finger. On the removal of the principal tumor, in order to destroy completely the numerous vegetations about its base, I found it necessary to remove the second incisor and its neighboring canine. With the scissors and the bistoury I could then excise all the suspected parts. An olive shaped cautery, heated to whiteness, was then passed backward and forward over the wound, so as to destroy not only the remaining portions of the morbid product, but also the periosteum. This little operation was well borne by the patient who lost at most 15 ounces of blood. Lotions and applications of cold water were immediately applied. No accident followed the operation. A slight inflammation followed by sloughing off the eschars formed by the cautery, took place in the next few days. Fleshy excrescences soon followed by a cicatrix were developed upon the spot where the tumor had been. The cure was delayed only by the presence of a little superficial necrosis of the maxilla. It was perfect about the first of March, the operation having been performed the 3d of February. A solid cicatrix of a mucous appearance covered the edge of the maxilla, and the general condition of the patient was much improved. Since then I have repeatedly seen the patient, and he has had no relapse. The removed tumor was very firm, the bistoury divided it with difficulty, its section of a pearly white, reddened in points, was brilliant, pressure caused no exudation of liquid more than the act of scraping with the bistoury; all its parts were exactly alike. The second by Rouillard, a carcinomaton epulis of the lower jaw, treated by excision. Recurrence—cauterization followed by cure.

A young lady eighteen years of age, of delicate constitution, formerly rachetic, had a fleshy excresence which grew from the internal face of the left side of the lower jaw, where it took root beneath the first and second molar, extended so far as the inner face of the right side. This tumor occupying almost the entire space of the arch of the lower jaw, had displaced from it the tongue, and kept it applied to the palate, so that the patient spoke, eat, and swallowed with difficulty. The upper surface of this fungosity somewhat resembling a great flattened horse chestnut, was half opened by a deep and irregular crevice whence issued a bloody oozing. Its pedicle was not larger than a twenty-four sous piece, and its mass was free and floating in the mouth. The patient suffered almost continued lancinating pains, which were often aggravated during the night, the interior of the bone seeming to be their principal seat. This tumor was three years old; its origin was refered to a laceration of the gums by a piece of nutshell. It would have been easy to apply the ligature to the tumor, but the surgeon after having extracted the first two molar teeth, which were very vacillating, thought proper to use the bistoury. The tumor was removed without difficulty, astringents and compression served to arrest the hemorrhage. On the next day, and for eight days the wound was cauterized with lapis infernalis, but the condition of the wound underwent no favorable change. Its repullation was so rapid that in the evening, it could not be perceived that the caustic applied in the morning had at all diminished the elevation of the flesh. It was always hard, unequal, painful and bleeding at the slightest touch. M. Brouillard then resorted to the actual cautery, (of silver). The fall of the eschar took place on the eighth day. It left a hollow surface without regerminating vegetations as before, still the aspect of the wound was not yet satisfactory; the bottom was hard and bleeding, slight lacinations were felt, and the fungous growths seemed ready to reappear. A second application of the cautery was considered the more necessary as it was evident that the roots of the disease were planted in the bone, and that it could not be destroyed without producing exfoliation. The eschar which succeeded this second application did not fall to the twelfth day, but the vicious local action was totally destroyed, the wound was covered with healthy granulations; the exfoliation of the bones took place almost insensibly, and the cure was perfect two months after the second application of fire. We will now give two cases of epulis removed by the third method.

Miss A., æt. 23, presented herself to us for the examination, and if possible permanent removal of a malignant epulis. She had had two operations performed, and after each one the tumor was reproduced after an interval of ten or twelve days. It was situated on the inside of the lower jaw of the left side, between the lateral incisor and first molar. It extended under the tongue towards the frenum, and between the teeth, so that a portion of it was on the outside. It rose above the grinding surfaces, as in eating, the jaws would close upon it. It was rather hard, in color red, very much pain accompanied it, being through the temple and ear. The teeth were not materially loosened. Her general health was being impaired, and we at once ordered tonics. It having appeared twice after removal, she was willing to have any thing done which effectually would remove the offending body. The books tell us to extract the teeth on each side and remove the piece of bone to which the tumor is attached. But in this case we disliked to produce such a deformity as would arise from that operation, and yet we were determined that the operation should be thorough. Finally, we decided to remove the tumor without the loss of the teeth. In December, 1861, we proceeded to operate. My father having with a bistoury, passed around the edge of the tumor, and then with a broad hoe-shaped scraping instrument, tore it with great force instantly from its attachment. Then with an instrument triangular in shape, very like a pickaxe, with the finger upon the teeth proceeded to remove the bone to which the tumor was attached, to the depth of from an eighth to a quarter of an inch, carefully touching every point. Between the teeth a V shaped instrument was used, taking off large pieces of bone. In fact, for a space larger than that covered by the tumor, the bone was removed to quite a depth, though the attachment of the teeth was not impaired. Having taken off, as we supposed, sufficient, we tied cotton on a small wire, this wet, was dipped in powdered nitrate of silver. This we passed over the wounded surface in every part for the space of twenty minutes, constantly replenishing the caustic. The mixture that we put on had the consistence of cream. It was necessarily a long and tedious operation, but the result of it completely justified us in it, for now, after a lapse of two years, no sign of a reproduction of the tumor has taken place, and yet the young lady retains her teeth, and there is no disfiguration. On the inside the teeth are denuded quite low down, but they are firm.

Case 2.—In October, 1862, I. M. B., æt. 40, consulted us in regard to an epulis, situated upon the lower jaw between the left canine and first molar. It was malignant, in size that of an almond shell, compressible. He suffered much pain from it. It had been removed three times, but always reappeared. The operation simply consisted in cutting off the fleshy mass. He was very desirous of having something done that would stay the progress of the disease. Having stated to him the probability of effecting a cure by a rather severe operation, I proceeded to operate in the same manner as in case No. 1. Although I think I removed more of the alveoli around the teeth than in the former case. In August saw the mouth, and there was no attempt at recurrence. The teeth are a little loose, but are not impaired for active service. The gums around the teeth are slightly reddened, and at times he has a little pain, but no unhealthy growth has made its appearance.

